# Corrosion‐Protected Hybrid Nanoparticles

**DOI:** 10.1002/advs.201700234

**Published:** 2017-09-15

**Authors:** Hyeon‐Ho Jeong, Mariana Alarcón‐Correa, Andrew G. Mark, Kwanghyo Son, Tung‐Chun Lee, Peer Fischer

**Affiliations:** ^1^ Max Planck Institute for Intelligent Systems Heisenbergstr. 3 70569 Stuttgart Germany; ^2^ Institute of Materials École Polytechnique Fédérale de Lausanne (EPFL) CH‐1015 Lausanne Switzerland; ^3^ Institute for Physical Chemistry University of Stuttgart Pfaffenwaldring 55 70569 Stuttgart Germany; ^4^ UCL Institute for Materials Discovery and Department of Chemistry University College London Christopher Ingold Building, 20 Gordon Street London WC1H 0AJ UK

**Keywords:** 3D core–shell nanoparticle, corrosion protection, hybrid nanocolloid, nanoscale encapsulation

## Abstract

Nanoparticles composed of functional materials hold great promise for applications due to their unique electronic, optical, magnetic, and catalytic properties. However, a number of functional materials are not only difficult to fabricate at the nanoscale, but are also chemically unstable in solution. Hence, protecting nanoparticles from corrosion is a major challenge for those applications that require stability in aqueous solutions and biological fluids. Here, this study presents a generic scheme to grow hybrid 3D nanoparticles that are completely encapsulated by a nm thick protective shell. The method consists of vacuum‐based growth and protection, and combines oblique physical vapor deposition with atomic layer deposition. It provides wide flexibility in the shape and composition of the nanoparticles, and the environments against which particles are protected. The work demonstrates the approach with multifunctional nanoparticles possessing ferromagnetic, plasmonic, and chiral properties. The present scheme allows nanocolloids, which immediately corrode without protection, to remain functional, at least for a week, in acidic solutions.

## 1 Introduction

**Figure 1:**
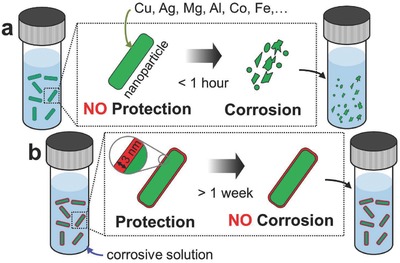
Corrosion‐resistant hybrid nanoparticles with a nm thick shell. a) Schematic of the corrosion process of the nanocolloids made of highly reactive materials in corrosive solution (e.g., acidic buffer, blood, H_2_O_2_, and even water). Without protection they corrode within hours. b) On the contrary, in the same solution, those grown and protected by the vacuum‐based growth scheme, which we present here and that involves deposition onto particles adhered to a surface, are stable against corrosion. The scheme is general and can accommodate hybrid nanoparticles with a variety of sizes (e.g., 8–200 nm),^16^ shapes,^[8a]^ and material compositions (Ag‐Cu,^37^ Ag‐Ti,^12^ Mg,^[27a]^ Ni,^38^ Au‐Fe,^11^ and here Cu and Co).

Corrosion is a ubiquitous characteristic of metallic solids in which a base metal is converted into its ionic state or oxide form by electrochemical surface reactions.^1^ Nanoparticles are especially vulnerable because of their high surface‐to‐volume ratios.^2^ In many cases, since the reaction products are soluble, corrosion leads to complete destruction of the original nanostructure (see **Figure** **1**a). This means that in real world applications the dominant materials consideration has often been corrosion stability, and the functional property required by the application, e.g., optical response, mechanical strength, magnetic susceptibility—is secondary. With effective corrosion protection materials selection can be made on the basis of the functional property. This will permit better materials optimization and lead to nanoparticles that are less expensive or more effective at the targeted application.

A rational approach to prevent the corrosion of a nanoparticle is to isolate it from the environment within an inert conformal shell (see Figure 1b). This is challenging however, as the protection layer must be stable in various environments and compatible with the nanoparticle itself, while simultaneously preserving the intended function of the nanomaterial. For instance, in plasmonic sensing applications, the near field sensing volume of a metal nanoparticle decays exponentially into the environment, so thick shell layers (>5 nm) reduce the effective sensing volume and efficiency.^3^ Moreover, magnetic particles intended for in vivo medical application must remain small and should not corrode.^4^ Encapsulating nanoparticles by chemical means has been proposed for protection, but in solution‐based processes the core material often oxidizes and corrodes during the coating process itself.^5^ It is also difficult to form a complete defect‐free shell with well‐defined thickness, since chemical coating methods typically require careful and laborious optimization whenever the core material or geometry is changed.^5^, ^6^ Flame‐based synthesis has also been proposed for the growth of core–shell nanoparticles.^7^ While the method does not permit the material composition or the shape of the core to be tuned, the carbon shell provides remarkable chemical stability.

Here, we report an approach for the design and fabrication of 3D core–shell nanoparticles and nanostructures with a nm thick shell that encapsulates the entire nanoparticle. A key element of the scheme is an inert protective segment, directly incorporated into the core's growth process that shields the particle's underside. We show that particles protected in this way are stable for days in acidic solutions in which in their unprotected state they dissolve in minutes. This work paves the way for the use of reactive, toxic, and unstable magnetic and plasmonic nanoparticles in physiological fluids.

## 2 Results and Discussion

Our method is based on a published wafer‐scale 3D nanofabrication scheme, which we call “nanoGLAD,”^8^ that combines block copolymer micelle nanolithography (BCML)^9^ with glancing angle deposition (GLAD).^10^” The method permits control over both the shape and material composition of 3D hybrid nanostructures that have been used as plasmonic nanoantennas for nanorheology^11^ and chiral sensing.^12^ However, many of the materials that can be grown with this technique are not chemically stable when exposed to air or water. This limits the scope of potential applications that require nanoparticles in a colloidal form.

Atomic layer deposition (ALD) is a promising general method for corrosion protection, since it is compatible with many materials and provides well‐controlled surface coatings.^13^ In contrast to chemical processes, one advantage of ALD is that, because it is a vacuum and gas phase technique, corrosion during the application process can be minimized. However, in a typical ALD process, nanoparticles are deposited onto a substrate for the coating step. This means that the undersides of the nanoparticles, which face the substrate, are not exposed to the reacting species in the ALD process.^14^ Thus the nanoparticles are not fully coated and their protective shells have defects. Once the nanoparticles are transferred to solution, corrosion will proceed via the defect which limits the lifetime of the nanomaterial. Thus, the practical challenge for the protection of nanoparticles via an ALD process is to ensure the complete encapsulation by a continuous shell layer without defects (see Figure S1, Supporting Information).^15^

**Figure 2:**
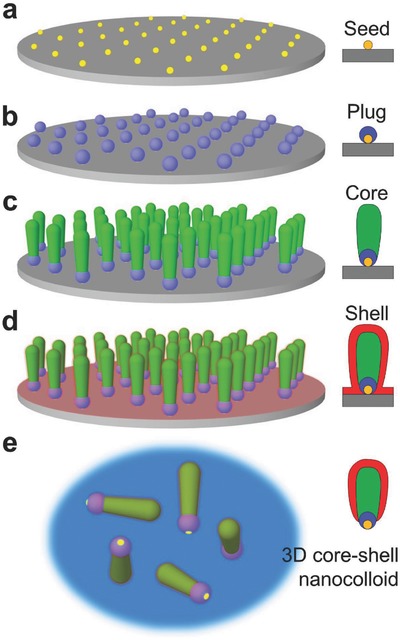
Fabrication of corrosion‐resistant 3D core–shell nanocolloids. a) Patterning a silicon wafer with self‐assembled Au nanodots using BCML. b) Growth of a protection patch, the “plug,” using GLAD. c) Growth of the “core”; the 3D functional nanostructures in need of protection. d) Formation of the second protection component, the “shell,” using ALD. e) A suspension of 3D core–shell nanoparticles in solution after detachment from the wafer by sonication.

To address this, we have developed the bottom‐up parallel fabrication scheme depicted in **Figure** **2**. BCML is used to spin‐coat an array of metallic nanoparticles with the desired particle diameter and spacing (here 10 and 100 nm, respectively) on a substrate such as a wafer (Figure 2a). The self‐assembled nanoparticles serve as nanoseeds for the subsequent vapor deposition of an inert “plug” segment. The plug is the crucial element that ensures that the bottom‐facing surface of the nanoparticle is protected (Figure 2b). The deposition of this small nanopatch is only possible with a shadow growth technique, as other methods would coat the entire surface and cannot deposit a material at defined positions.^16^ It is important that the material of the plug is stable in solution, ideally under a wide range of chemical conditions. We find that metals which form tenacious metal oxides, particularly Ti, act as good plugs (Figure S2, Supporting Information). Next, the functional, but reactive “core” of the nanoparticle is grown on top of the plug by further nanoGLAD deposition (Figure 2c).[[qv: 8a,b]] Many core shapes are readily obtained using this technique (see our earlier report in ref. [[qv: 8a]] for further details), but here we focus on protecting two representative shapes: nanorods and nanohelices. In a final step, the core is encapsulated by ALD, which forms a thin and chemically inert oxide “shell” over the exposed surface of the nanostructures (Figure 2d).^17^ Here we use HfO_2_ and Al_2_O_3_, but other inert and ALD‐compatible materials including TiO_2_, SiO_2_, ZnO, Pt, etc., could be also used for protection.^18^ The ALD layer covers the core nanomaterial and overlaps the plug; together the plug and shell ensure complete protection of the core from chemical attack at all points. Finally, depending on the application, the protected functional nanoparticles can be released into solution by sonication (Figure 2e). The advantage of our scheme is that it is general and that it readily accommodates changes in the shape and material composition of the hybrid nanocolloids. We now show how Cu and Co nanocolloids grown using physical vapor deposition can be stabilized such that they remain functional in a variety of aggressive aqueous solutions for many days.

**Figure 3:**
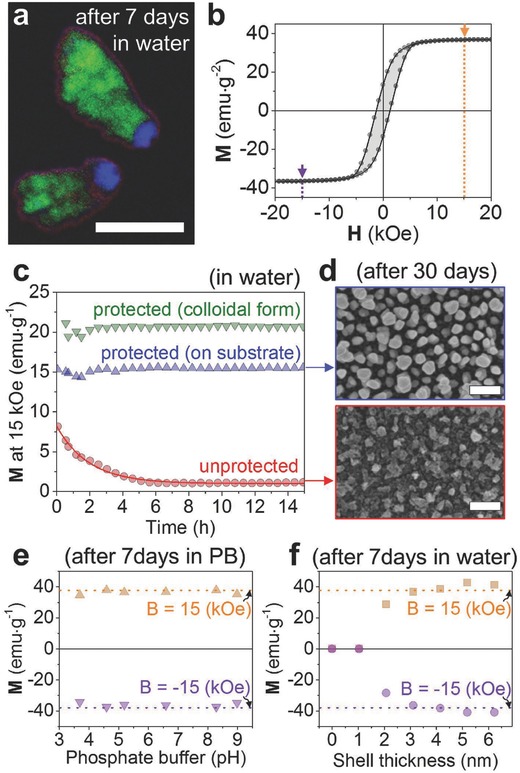
Protection of Co nanorods. a) EF‐TEM elemental map of the protected Co nanorods after 7 d immersion in water (green: Co, blue: Ti, red: O, scale bar: 100 nm). b) Out‐of‐plane magnetization measurement of Co nanorods as‐grown on the Si substrate, showing an open ferromagnetic hysteresis loop. The arrows indicate the saturation magnetization of Co nanorods (at ± 15 kOe), whose values were used to track the stability of the nanorods. c) In situ trace in saturation magnetization of the Co nanorods: In‐plane saturation magnetizations of the protected (blue, up triangle) and unprotected (red, circle) Co nanorods on‐water at H = 15 kOe in water as a function of time (see Figure S9a, Supporting Information). The unprotected particles had decayed substantially in the short amount of time between preparation and measurement. The solid red line was fitted by using first‐order reaction and the decay rate of *k* is 142 × 10^−6^ s^−1^. Saturation magnetization of the protected Co nanorods suspended with the isotropic orientation in 2% agarose gel (green, down triangle) at H = 15 kOe in water as a function of time (see Figure S9b, Supporting Information). d) SEM images of the protected Co nanorods (top panel) and the unprotected Co nanorods (bottom panel) after 30 d in water. e) Out‐of‐plane saturation magnetizations of the Co nanorods protected with 4 nm thick HfO_2_ after 7 d in 0.1 m phosphate buffer solutions at 6 different pH conditions (pH 3.7–pH 9). The dotted lines indicate the positive and negative saturation magnetizations of the particles as grown. f) Out‐of‐plane saturation magnetization of Co nanorods protected with different thicknesses of HfO_2_ layer at H = 15 kOe (orange) and −15 kOe (violet) after 7 d in water.

Magnetic and metallic nanoparticles are of interest for medical applications ranging from sensing^4^, ^19^ to therapy,^20^ but often the optimal materials for a given application cannot be used for reasons of physiological incompatibility. For instance, hard magnetic materials are often toxic and many metals such as copper or silver corrode easily. We demonstrate here our protection scheme with cobalt, a strong ferromagnetic material, but one that is not chemically stable in solution when grown in the form of nanoparticles using physical vapor deposition.[[qv: 7a]] **Figure** **3**a shows two Co nanorods protected with ≈20 nm Ti plugs and 4 nm thick HfO_2_ shells (see Figures S3–S6 for other ALD conditions, i.e., thickness and material, and Figure S7 for images showing a large number of particles, Supporting Information). The energy filtered transmission electron microscope (EF‐TEM) image, acquired after 7 d immersion in water, clearly shows that the Ti plug (blue) and HfO_2_ shell (red) combine to cover the entire surface of the reactive Co core (green). Even after 30 d, we did not observe corrosion of the nanoparticles (see Figure 3d; and Figure S6, Supporting Information). Figure 3b shows the out‐of‐plane ferromagnetic character of Co nanorods measured on the wafer before exposure to water. In this geometry the long axis of the particles is parallel to the applied magnetic field direction and the sample exhibits a coercive field of ≈1 kOe and a remanence of ≈14 emu g^−1^ (see SQUID magnetometry in the Experimental Section for details). The effectiveness of the protection can be assessed by monitoring the saturation magnetization (B = 15 kOe) after immersion in water. Figure 3c shows the evolution of the saturation magnetization for three different samples: unprotected (red circles) and protected (blue triangles) particles on‐wafer, immersed in water with the nanorod axes normal to the applied magnetic field (see Figure S9a, Supporting Information), and protected particles with isotropic orientations in an agarose gel stabilized as colloidal suspension (green triangles, see Figure S9b, Supporting Information). The gel matrix traps the particles and prevents their aggregation and sedimentation over long times (at least several days) but is highly permeable to solvent and ions.^21^ Upon immersion, the magnitude of the signal for the unprotected particles is strongly attenuated, whereas both protected samples show no substantial change in signal over the course of the experiment (see also Figure S10 for details, Supporting Information). The behavior of the unprotected particles is well described by first‐order kinetics and yields an unprotected nanoparticle half‐life of 81 min once the finite magnetization of the Co ion products is considered (see Section 6, Supporting Information). The asymptotic magnetization of ≈1 emu g^−1^ is consistent with complete conversion of metallic Co to Co^2+^ which is expected to have a magnetization of ≈2 emu g^−1^ at 15 kOe.^22^ Scanning electron microscopy (SEM) images of the two on‐wafer samples acquired after 30 d of immersion (Figure 3d) show strong degradation of the unprotected structures (lower) but the protected structures appear essentially unaffected (upper).

For biomedical applications the nanoparticles must be chemically stable in solutions with a variety of ion concentrations and pH values. Figure 3e shows the saturation magnetization of protected on‐wafer Co particles after 1 week in 0.1 m phosphate buffer solutions in the range of pH 3.7–pH 9.^23^ Both the SEM images (Figure S11, Supporting Information) and the SQUID measurements demonstrate that the protected hybrid nanocolloids are stable even in highly acidic solutions (Figure S12, Supporting Information).

The thickness of the HfO_2_ shell is an important factor in the degree of protection. Figure 3f shows saturation magnetizations for on‐wafer particles with shell thickness ranging from 0 (no shell protection) to 6.25 nm after 1 week in water (see also Figure S8, Supporting Information). Oxide layer thickness less than 3 nm offer poor protection but at shell thicknesses ≈3 nm and greater the magnetization is unchanged even after 7 d immersion. These results are in excellent agreement with the SEM analysis shown in Figure S6 (Supporting Information).

**Figure 4:**
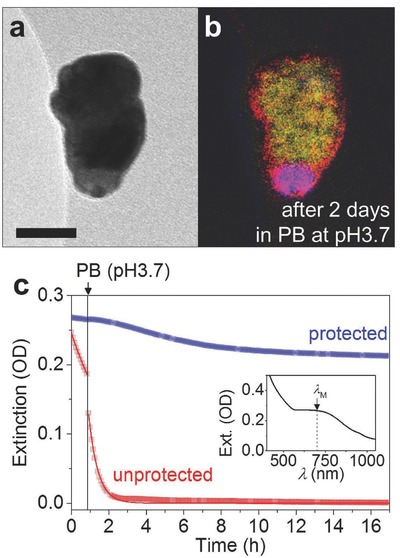
Protection of Cu nanorods. a) TEM of the Cu nanorod protected with 3 nm HfO_2_ layer and b) its corresponding EF‐TEM elemental map after 2 d immersion in 0.1 m PB at pH3.7 (yellow: Cu, blue: Ti, red: O, scale bar: 50 nm). c) In situ extinction trace (at λ = 687 nm) of the protected Cu nanorods (blue) and the unprotected Cu nanorods (red) suspended in agarose matrix. Initially, the liquid phase of the gel is water; the vertical line indicates the addition of acidic phosphate buffer which after mixing yields a concentration of 0.1 m at pH3.7. Each solid line was fitted by using first‐order reaction, which yields the decay rates of *k* = 92 × 10^−6^ s^−1^ (water) and *k* = 563 × 10^−6^ s^−1^ (PB). Inset shows the extinction spectrum of the protected Cu nanorods in 2% agarose gel.

Au is probably the most commonly used material in the nanoparticle field because of its exceptional stability and useful plasmonic properties.[[qv: 20a,24]] However, other metallic materials (including Cu,^25^ Al,^26^ and Mg^27^) are often overlooked, despite favorable optical properties, due to their inherent instability in air and water. In particular, Cu is highly conductive, and could represent a cost‐effective replacement for Au in plasmonic, photovoltaic, and catalytic applications,^28^ but its use is limited as it oxidizes quickly.[[qv: 25a]] As a second demonstration, we introduce Cu nanorods protected with a ≈20 nm Ti plug and 3 nm HfO_2_ shell (see Figure S13 for more images of different ALD thicknesses, Supporting Information). **Figure** **4**a,b shows TEM and EF‐TEM images of a single such nanorod after immersion in phosphate buffer solution (0.1 m, pH 3.7) for 2 d (Figure S14 shows a large number of particles, Supporting Information). It can be clearly seen that the Cu core remains uncorroded thanks to the plug and shell protection.

Part of the appeal of Cu for nanoparticles is that it is a low‐cost material that supports a local surface plasmon resonance (LSPR) observable in optical extinction spectroscopy (Figure 4c, inset).^29^ Figure 4c shows the time evolution of the LSPR response of unprotected (red) and protected (blue) Cu nanorods dispersed with the isotropic order in a 2% agarose gel matrix. After ≈1 h of immersion, phosphate buffer (PB) solution was added to the gel and allowed to mix by diffusion (see the Experimental Section for details). Again the signal from the unprotected particles shows a strong decay with a half‐life of 125 min before PB addition and 22 min after. Within 3 h the signal has reduced to <2%, indicating almost complete dissolution of the Cu core (Figure S15 for the full extinction spectra, Supporting Information). On the other hand, the protected particles show only a small (0.7%) decrease in intensity during the initial phase,^30^ followed by a slow asymptotic decline after the PB is added to a steady state value of ≈78%. We speculate that the decay observed for the protected particles is due to a small fraction of colloids whose protective shell was damaged by sonication during the lift‐off process. This is supported by the observation that protected Cu particles left on‐wafer remained uncorroded after 2 d immersed in the same buffer solution (see TEM images in Figure S14, Supporting Information).

**Figure 5:**
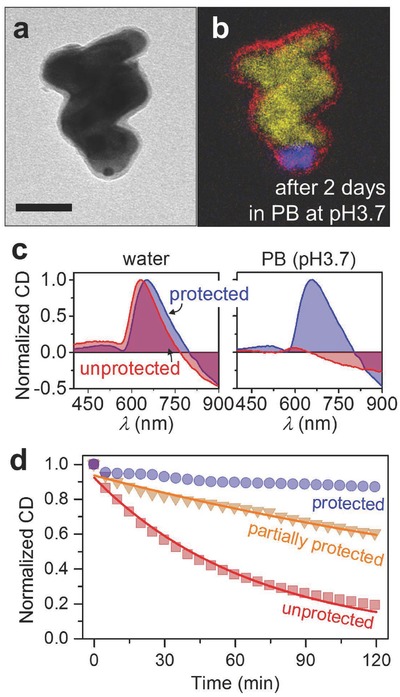
Protection of helix‐shaped Cu nanoparticles. a) TEM of the Cu nanohelix protected with 3 nm HfO_2_ layer and b) its corresponding EF‐TEM elemental map after 2 d immersion in 0.1 m PB at pH3.7 (yellow: Cu, blue: Ti, red: O, scale bar: 50 nm). c) The normalized CD spectra of the protected Cu nanohelices (blue) and the unprotected Cu nanohelices (red) in water (left panel) and 10 × 10^−3^ m phosphate buffer at pH 3.7 (right panel, see also Figures S22 and S23, Supporting Information). For the PB results, the unprotected particles had decayed substantially in the short amount of time between preparation and measurement. d) Comparison of the stabilities of the Cu nanohelices that are completely protected (blue), partially protected (orange), and unprotected (red) in 10 × 10^−3^ m H_2_O_2_ by tracking their CD intensity with time. Each solid line was fitted by using first‐order reaction, which yields the decay rates of *k* = 63 × 10^−6^ s^−1^ (orange; partially protected) and *k* = 250 × 10^−6^ s^−1^(red; unprotected).

A particularly sensitive probe of the structure and symmetry of nanocolloids is circular dichroism (CD) spectroscopy. We can grow chiral nanoparticles by simply changing the growth parameters in the nanoGLAD fabrication step.^12^ Here we grow Cu nanohelices encapsulated by a ≈20 nm Ti plug and 3 nm HfO_2_ shell, see **Figure** **5**a,b (see also Figures S16 and S19 for other ALD conditions, Supporting Information). The left panel of Figure 5c shows the corresponding normalized CD spectra for protected and unprotected particles immediately after immersion in water. The spectral peak of the protected particle is redshifted relative to that of the unprotected one because the plasmon extinction spectrum is sensitive to the refractive index of the local environment, and the index of HfO_2_ is larger than that of the water it displaces. Then, to test the particles' stability in acidic solution, we added phosphate buffer which yielded a concentration of 10 × 10^−3^ m PB solution at pH 3.7 after mixing. In this case, as shown in the right panel of Figure 5c, the signal of the unprotected particle has diminished in the short period of time between mixing the solution and making the measurement (<4 min, Figure S22, Supporting Information). The strong attenuation of the peak is consistent with a population of fewer and smaller metallic Cu nanohelices. Corresponding dynamic light scattering (DLS) studies show that the protected Cu nanoparticles' size distribution is stable, whereas the unprotected colloids reveal no meaningful features (Figure S22, Supporting Information).^31^ After 20 min the signal from the unprotected sample is indistinguishable from baseline noise, whereas the protected sample, after 5 d, retains 50% of its original intensity (see Figure S23, Supporting Information). SEM and TEM images show that the protected nanocolloids remain intact after at least 2 d immersion (Figure S20 for SEM images and Figure S21 for TEM image, Supporting Information).

We also measured in detail the time evolution of protected (plug and shell, Figure 5d, blue), partially protected (shell only, yellow), and unprotected (without plug and shell, red) Cu nanohelices, by monitoring their CD response. When immersed in a 10 × 10^−3^ m H_2_O_2_ solution, the unprotected sample shows a ≈81% drop in CD intensity over 120 min, while the signal from the protected sample decreases only ≈13% in the same (see Figure S18 for complete CD spectra, Supporting Information). The small decline seen by the protected particles is attributed to those that are mechanically damaged during release from the wafer. Interestingly, the partially protected sample, which lacks a protective plug, showed signal attenuation midway between the protected and unprotected samples (40% drop). This clearly illustrates that the ALD shell alone is not sufficient for particle protection, and that the underside plug is a crucial feature for long‐term nanoparticle protection in corrosive environments.

## 3 Conclusion

In summary, we have shown that nanoparticles can be effectively protected against corrosion by combining a physical vapor shadow grown plug with an atomic layer deposited shell. The method permits the complete encapsulation of the nanoparticle and we have demonstrated stabilities lasting from a week to over a month without loss of magnetic or plasmonic functionality. The stability could be shown even when the nanoparticles are immersed in physiological buffers, acidic environments, and reactive solutions. We have shown protection of both nanorods and nanohelices of Co and Cu, and expect that the scheme can be extended to produce corrosion resistant nanoparticles with the full range of core shapes^12^, ^32^ and compositions[[qv: 8a,11,12]] that nanoGLAD^14^ is capable of. This opens up a number of potential applications for nanoparticles composed of materials that are, at present, considered too reactive or toxic for practical use. These include unstable magnetic and plasmonic materials as nanoparticles for sensing,[[qv: 19a,27a]] imaging,[[qv: 19b,c,33]] as catalysts,^5^, ^34^ and for biomedical applications.^4^, ^11^, ^35^

## 4 Experimental Section

*Block Copolymer Micelle Nanolithography*: The array of Au nanoseeds was prepared using block‐copolymer micelle nanolithography as previously reported.^9^ Briefly, the block‐copolymer micelles were formed by self‐assembly and then spin‐coated onto a 2 in. Si wafer where the micelles form a quasihexagonally ordered monolayer (spacing ≈100 nm). Plasma treatment reduced the Au to form metallic nanoparticles with ≈10 nm in diameter. These acted as seeds for subsequent nanoGLAD growth.

*Nano Glancing Angle Deposition*: nanoGLAD was used for two main steps, growing the plug and the nanoparticle core. In this process, a substrate manipulator provided independent control over the azimuthal ϕ, and polar α angles of the vapor flux during growth, within a chamber with a base pressure of 1 × 10^−6^ mbar. To form well‐isolated plugs, the flux angle α and the azimuthal rotation rate per unit thickness d*ϕ/*dθ were kept to, respectively, 85^o^ and 36 ± 0.1^o^ nm^−1^ with closed‐loop feedback based on measurement of the material deposition rate on a quartz crystal monitor. The core material was grown on the plugs, where d*ϕ/*dθ was maintained at a constant 18 ± 0.1^o^ nm^−1^ for Co (and Cu) nanorod and 1.8 ± 0.1^o^ nm^−1^ for Cu nanohelix, respectively, while keeping α = 87^o^.

*Atomic Layer Deposition*: A thin layer of alumina (Al_2_O_3_) or hafnium dioxide (HfO_2_) was grown to cover the core's surface using atomic layer deposition (Savanah 100, Cambridge Nanotech) using the standard conditions provided by the manufacturer (resulting in a growth rate of 0.1 nm cycle^−1^). The Al_2_O_3_ was grown at *T* = 100 °C with a 0.1 nm cycle^−1^ growth rate by injecting trimethylaluminum for 0.03 s and H_2_O for 0.03 s repeatedly. The HfO_2_ was grown at *T* = 150 °C with a 0.1 nm cycle^−1^ growth rate by injecting Hf(NMe_2_)_4_ for 0.15 s and H_2_O for 0.015 s, repeatedly.

*SEM, TEM, and EF‐TEM Analysis*: Structures were imaged with SEM (Gemini Ultra55, Zeiss), TEM (CM200, Philips), STEM (SESAM, Zeiss), energy dispersive x‐ray spectroscopy (EDX), and EF‐TEM. TEM samples were prepared by drop casting ≈20 μL of the colloidal suspension onto a Holey carbon‐coated TEM grid (Cu or Ni 400 mesh), followed by drying under a gentle flow of Ar gas. Bright field and EF‐TEM images were recorded on a Zeiss 912 Omega electron microscope under an accelerating voltage of 120 kV. For the EF‐TEM images the in‐column electron energy filter was used. The exposure time ranged from 5 to 30 s for the different samples and materials. Each elemental core‐loss image was measured, modeled, and subtracted by the “three‐window technique” using the power law model in the Gatan Digital Micrograph program (http://www.gatan.com/software/).

*SQUID Magnetometry*: The magnetization of the Co nanorods was measured at 300 K using a Quantum Design magnetic property measurement system magnetometer. For the analysis of the on‐wafer nanorods' stability in water, a small piece (≈10 mm^2^) of the Si wafer on which the nanorods were grown was immersed in water contained in a quartz NMR tube and sealed with a rubber cap (see Figure S9, Supporting Information). The *M–H* curves were corrected for the diamagnetism of the Si substrate by subtracting a linear background whose slope matches the high field diamagnetic response of the Si beyond the Co saturation magnetization.

*Colloids and Buffer Solution*: The nanoparticles were detached from the wafer by sonicating a wafer piece in an aqueous solution of 0.1 mg mL^−1^ polyvinylpyrrolidone for ≈2 min. The phosphate buffer was prepared using phosphate salt pairs, (Na_2_HPO_4_–KH_2_PO_4_), as well as NaCl and KCl. The pH for each solution was adjusted when necessary with HCl. Buffers were prepared as stock solutions and stored at 4 °C until further use.

*Agarose Gels*: For measurements on colloidal suspensions, agarose gel was used to prevent agglomeration of the particles during the long measurement.[[qv: 21a]] 4% agarose gel was melted at 100 °C then mixed at 1:1 with an aqueous colloidal suspension of the nanoparticles. The mixture, with 2% agarose concentration, was cooled in a fridge to form a gel. For the experiment described in Figure 3c, concentrated PB was added above the gel in sufficient volume that after diffusive dilution with the water phase of the gel the PB concentration was 0.1 m at pH 3.7.

*UV–vis–NIR Spectroscopy and CD Spectroscopy*: Extinction spectra of the nanocolloids were measured on a Cary UV–vis–NIR 5000 spectrophotometer. Circular dichroism spectra were obtained with a Jasco J‐810 circular dichroism spectrometer. Samples of nanoparticles trapped in agarose matrix were contained in quartz cuvettes with 10 mm path length. All the spectra were measured with a scan rate of 500 nm min^−1^ in the wavelength range of 400–1000 nm at 0.1 nm intervals. Extinction results were baseline corrected by subtracting a spectrum acquired from a glass cuvette containing PB solution and agarose gel.

*DLS Analysis*: A colloidal solution (0.5 mL) of nanohelices was measured in a Malvern Zetasizer Nano ZS at 2 min intervals. For the analysis of the Cu nanoparticles the refractive index and extinction of Cu at λ = 632.8 nm (He‐Ne laser) were taken from ref. 36.

## Conflict of Interest

The authors filed two patent applications related to the fabrication method.

## Supporting information

Supplementary
