# Supplementary material for: Corrosion‐Protected Hybrid Nanoparticles
Source: Adv Sci (Weinh). 2017 Sep 15;4(12):1700234. doi: 10.1002/advs.201700234 (PMC5737107; doi:10.1002/advs.201700234)
Supplement: Supplementary file 1 — Supplementary [file ADVS-4-na-s001.pdf]

Copyright WILEY-VCH Verlag GmbH & Co. KGaA, 69469 Weinheim, Germany, 2017.

Supporting Information

for *Adv. Sci.,* DOI: 10.1002/advs.201700234

Corrosion-Protected Hybrid Nanoparticles

*Hyeon-Ho Jeong, Mariana Alarcón-Correa, Andrew G. Mark, Kwanghyo Son, Tung-Chun Lee, and Peer Fischer**

Copyright WILEY-VCH Verlag GmbH & Co. KGaA, 69469 Weinheim, Germany, 2013.

Supporting Information

Corrosion-protected hybrid nanoparticles

Hyeon-Ho Jeong[1,2], *Mariana Alarcón-Correa*[1,3], *Andrew G. Mark*[1], *Kwanghyo Son*[1], *Tung-Chun Lee*[1,4], *and Peer Fischer*[1,3,]*

((Optional Dedication))

H.-H. Jeong, M. Alarcón-Correa, Dr. A.G. Mark, K. Son, Dr. T.-C. Lee, Prof. Dr. P. Fischer
[1]Max Planck Institute for Intelligent Systems, Heisenbergstr. 3, 70569 Stuttgart, Germany
E-mail: fischer@is.mpg.de

H.-H. Jeong
[2]Institute of Materials, École Polytechnique Fédérale de Lausanne (EPFL), CH-1015 Lausanne, Switzerland

M. Alarcón-Correa, Prof. Dr. P. Fischer
[3]Institute for Physical Chemistry, University of Stuttgart, Pfaffenwaldring 55, 70569 Stuttgart, Germany

Dr. T.-C. Lee
[4]UCL Institute for Materials Discovery and Department of Chemistry, University College London, Christopher Ingold Building, 20 Gordon Street, London WC1H 0AJ, United Kingdom

Keywords: nanoscale encapsulation, 3D core-shell nanoparticle, hybrid nanocolloid, corrosion protection

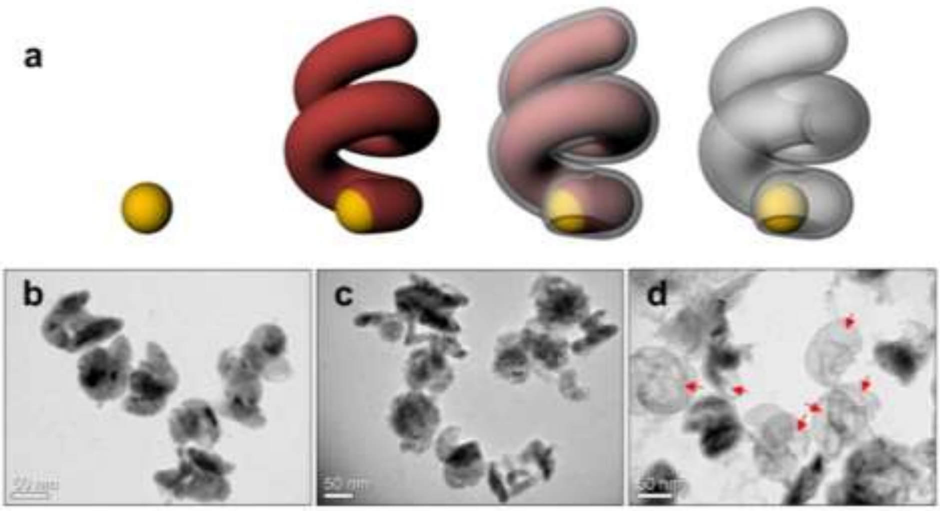

Figure S1. Protection of Cu nanohelices without plugs. a) Schematic view of the fabrication steps: Au nanoseed patterned by BCML, Cu nanohelix grown upon the seed by GLAD, shell formation by ALD, and nanohelix after corrosion of Cu (from left to right). TEM images of the Cu nanohelices protected with 3 nm thick Al_2O_3 layer after b) 5 min, c) 200 min, and d) 22 h in 15 mM H_2O_2. Red arrows indicate the helical shells remained after Cu corrosion.

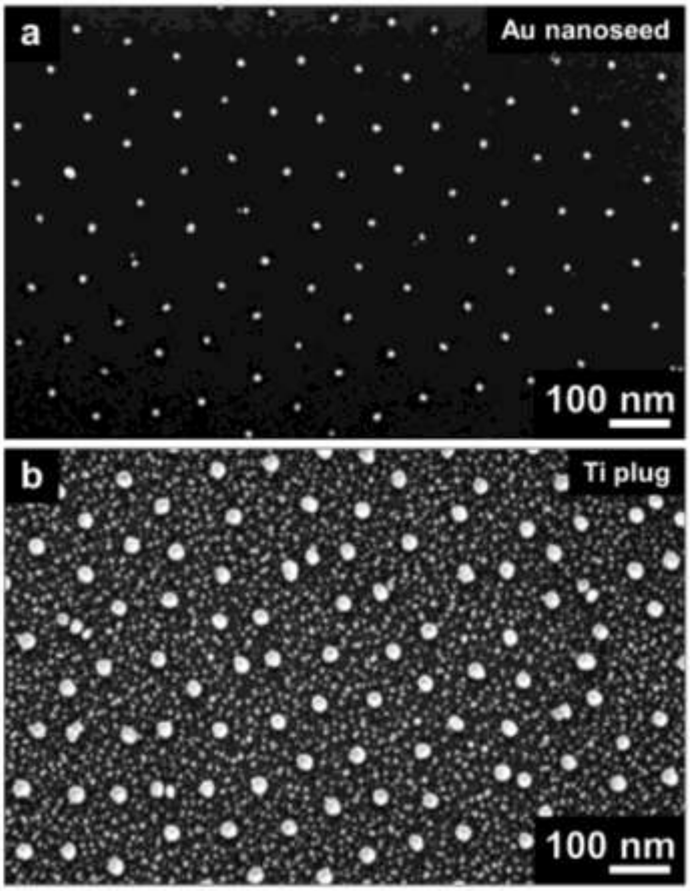

Figure S2. a) SEM image (top view) of an array of ~10 nm gold nanoparticles prepared by BCML. b) SEM image of the well-isolated plugs (Ti) on top of the Au seeds grown by GLAD.

1. Co nanorods protected with Al_2O_3

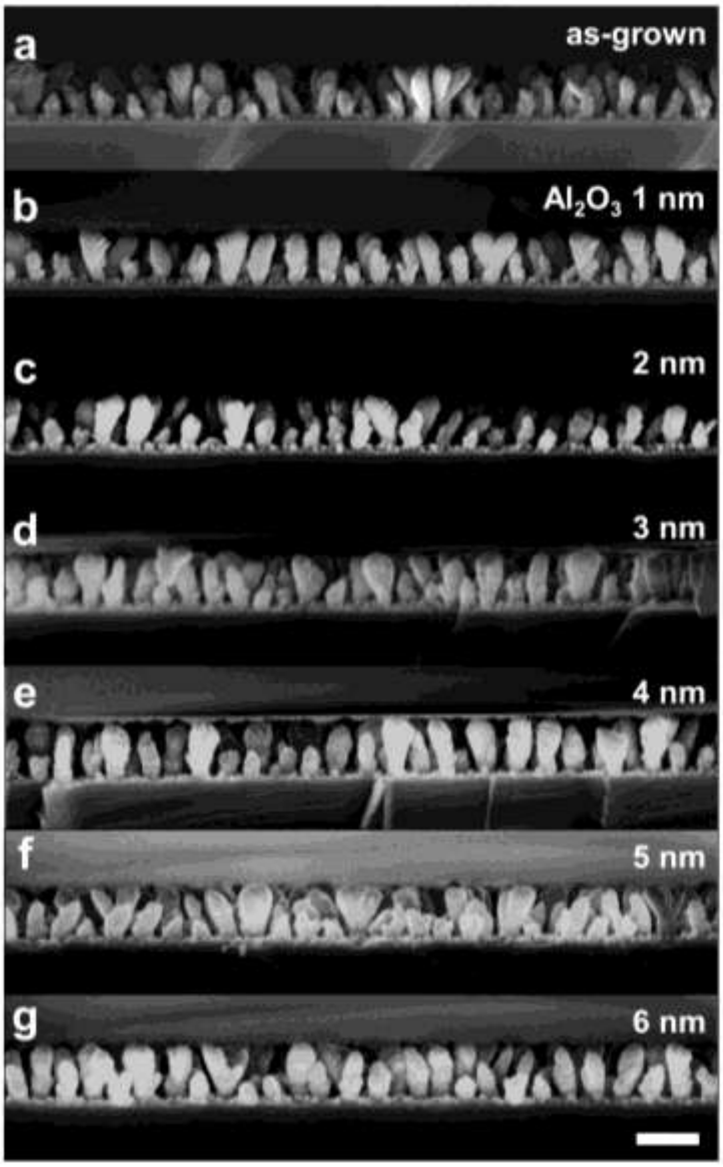

Figure S3. SEM images (side view) of the Co nanorods protected with different thicknesses of the Al_2O_3 shell layer: a) 0 nm, b) 1 nm, c) 2 nm, d) 3 nm, e) 4 nm, f) 5 nm, and g) 6 nm (Scale bar: 200 nm).

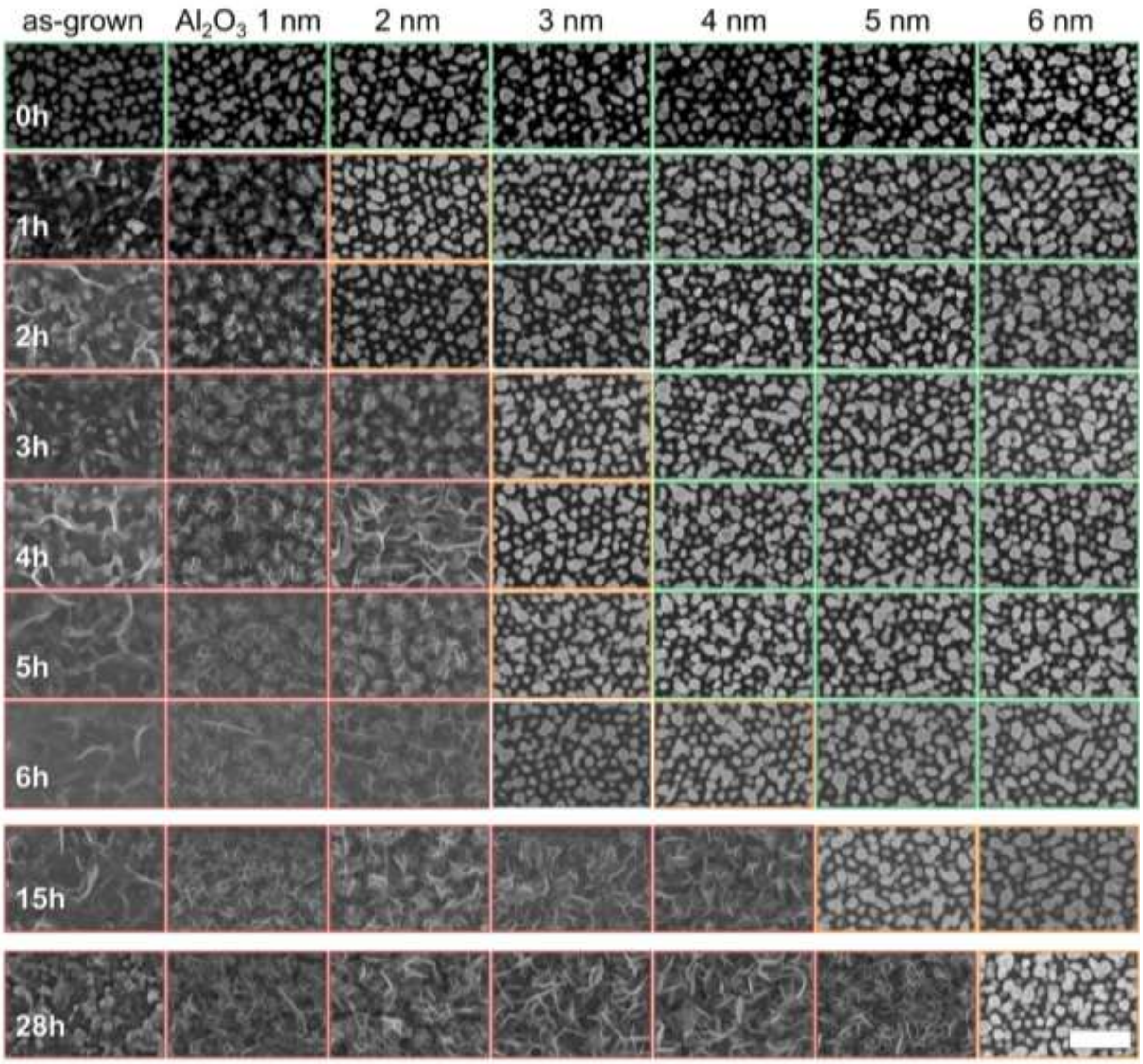

Figure S4. Serial SEM images (top view) of Co nanorods protected with an Al_2O_3 shell layer after immersion in water over time (Scale bar: 400 nm). Each column indicates a different ALD condition (0 to 6 nm thick Al_2O_3 layer from left to right). Each row indicates how long the particles were kept in water before imaging. The different boundary colors of each image indicate the status of the particles stability (red: complete corrosion, orange: partial corrosion, green: stable).

2. Co nanorods protected with HfO_2

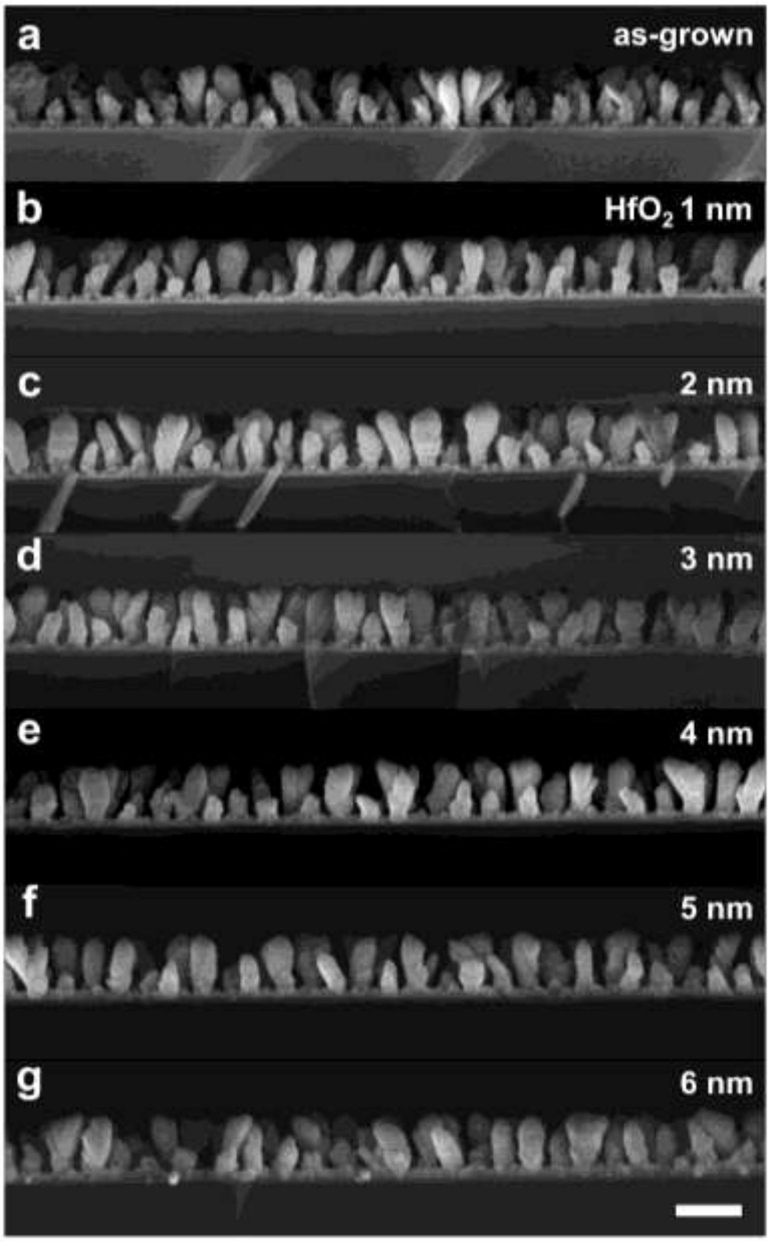

Figure S5. SEM images (side view) of the Co nanorods protected with different thicknesses of the HfO_2 shell layer: a) 0 nm, b) 1 nm, c) 2 nm, d) 3 nm, e) 4 nm, f) 5 nm, and g) 6 nm (Scale bar: 200 nm).

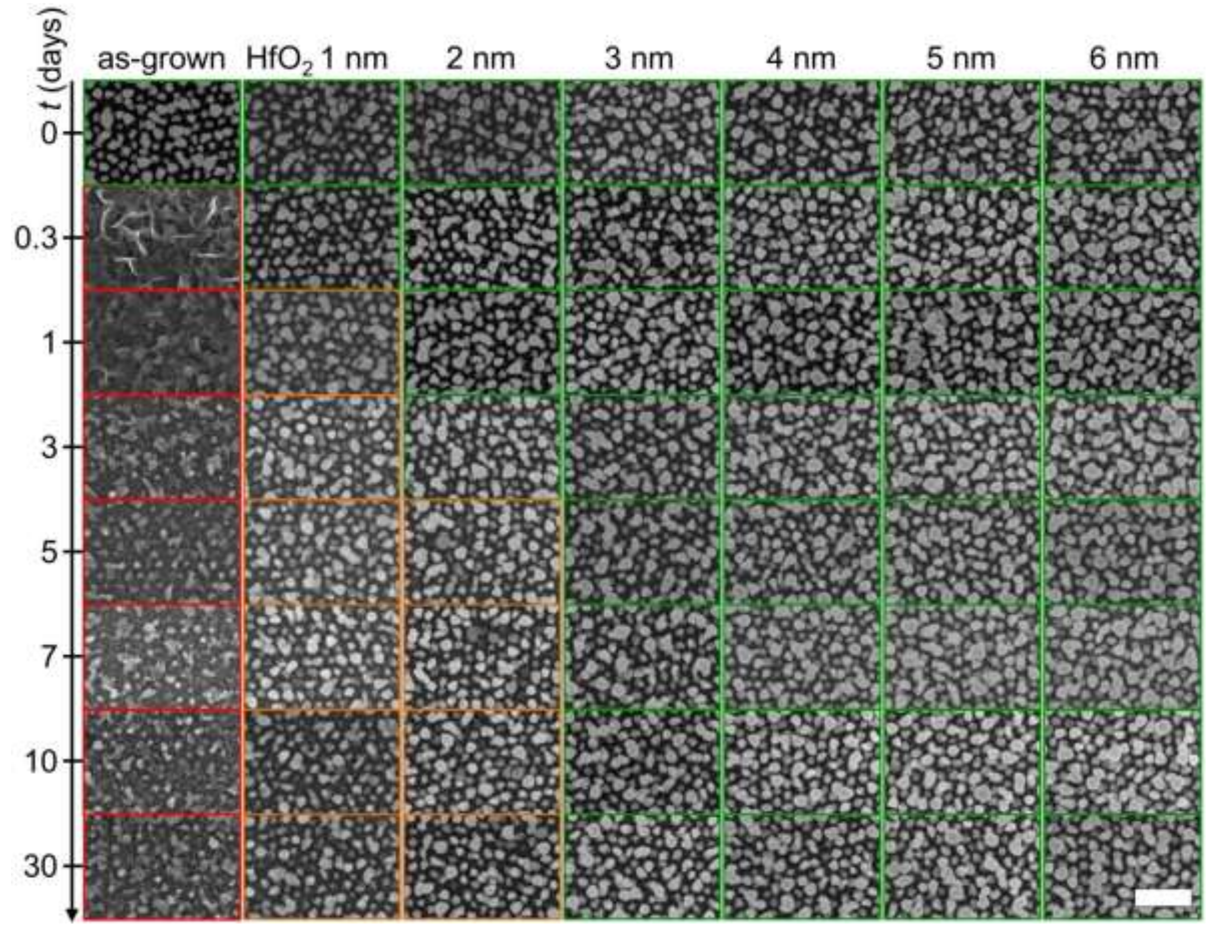

Figure S6. Serial SEM images (top view) of the Co nanorods protected with HfO_2 shell layer in water over time (Scale bar: 400 nm). Each column indicates the different ALD conditions (0 to 6 nm thick HfO_2 layer from left to right). Each row indicates how long the particles kept in water before imaging. The different boundary colors of each image indicate the status of the particles stability (red: complete corrosion, orange: partial corrosion, green: stable).

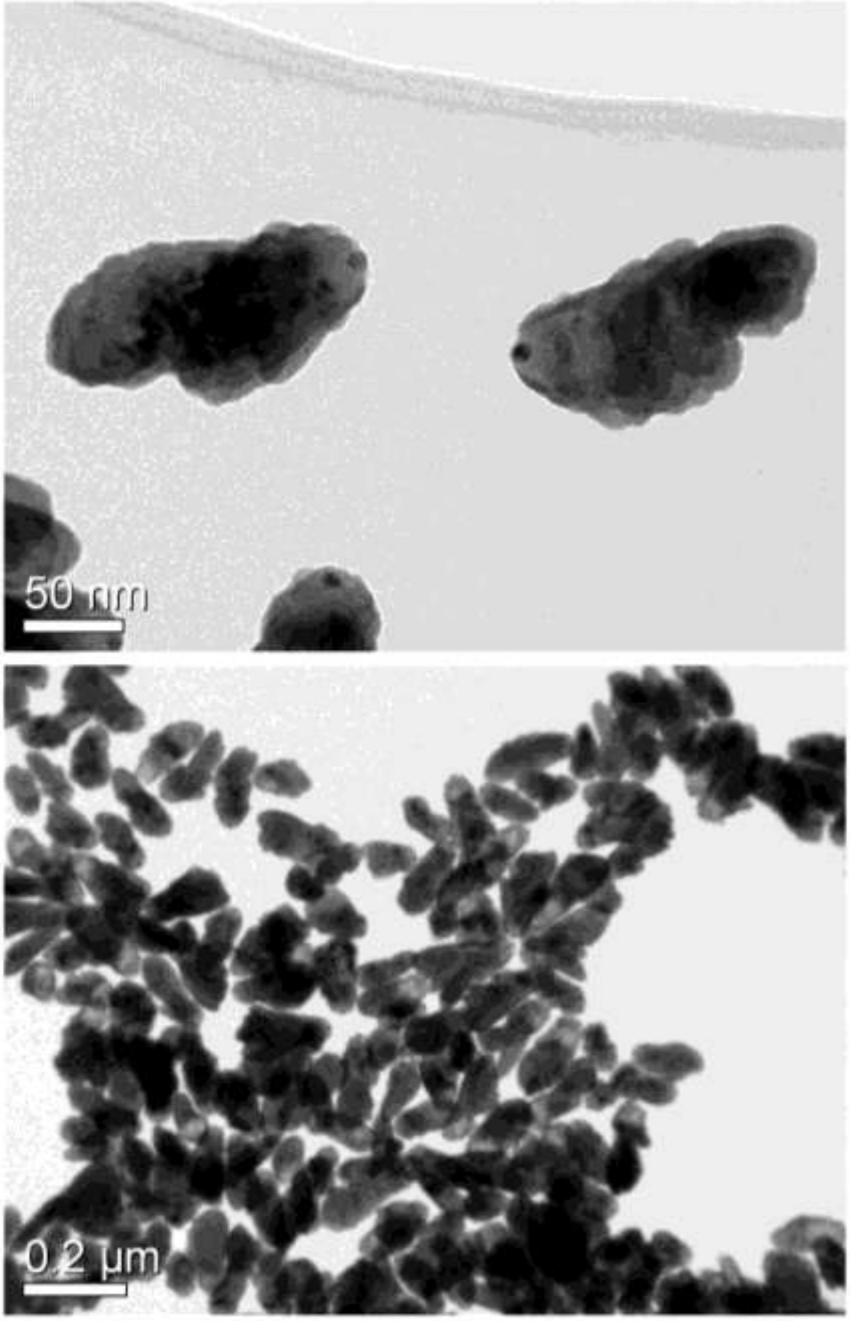

Figure S7. TEM images of the Co nanorods protected with 4 nm thick HfO_2 layer after 7 days in water.

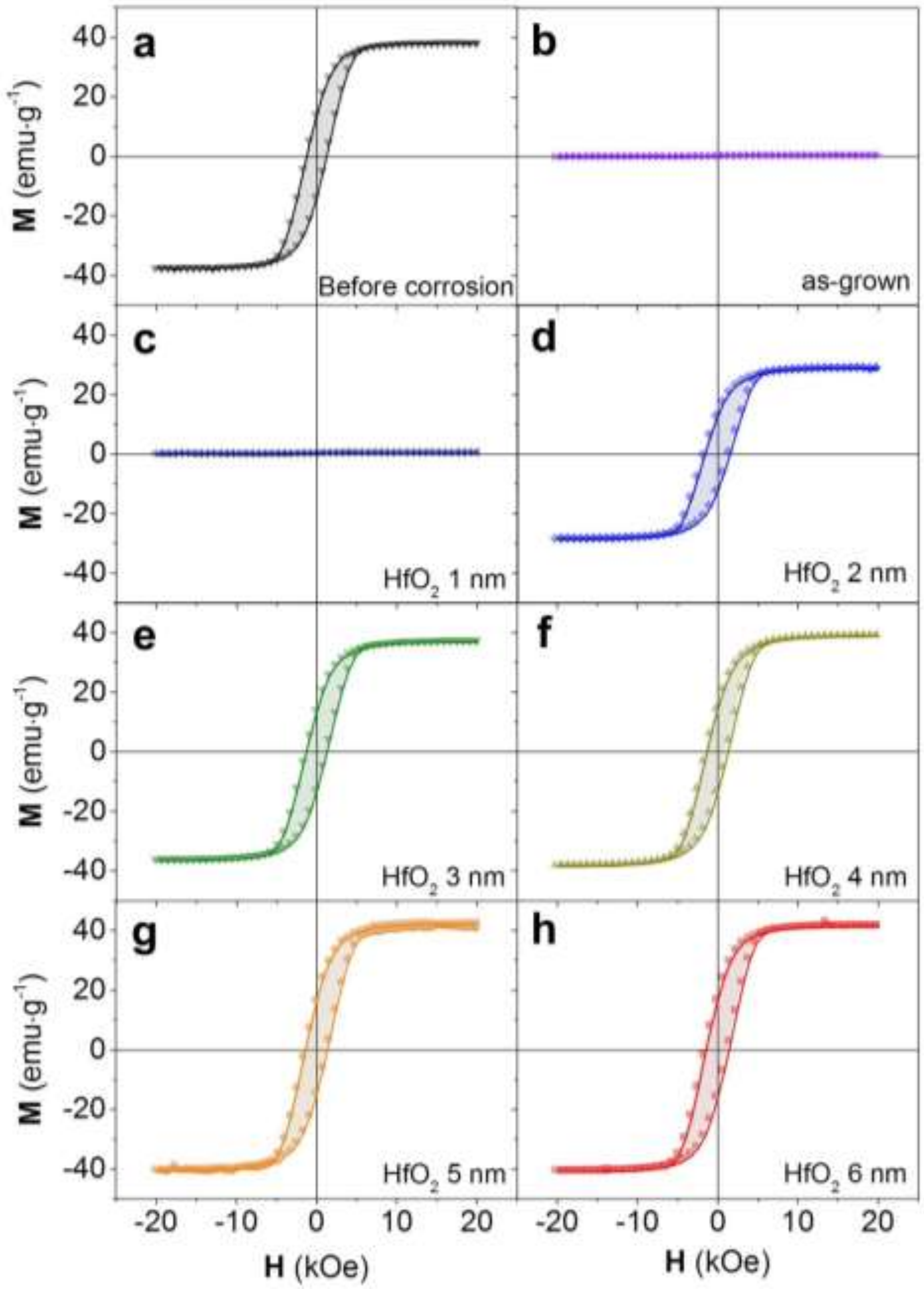

Figure S8. Our-of-plane ferromagnetic properties of the Co nanorods protected with different thicknesses of the HfO_2 layer on the silicon wafer: 0 nm a) before and b) after exposure to water (corrosion) for 7 days, and c) 1 nm, d) 2 nm, e) 3 nm, f) 4 nm, g) 5 nm, and h) 6 nm after exposure to water for 7 days.

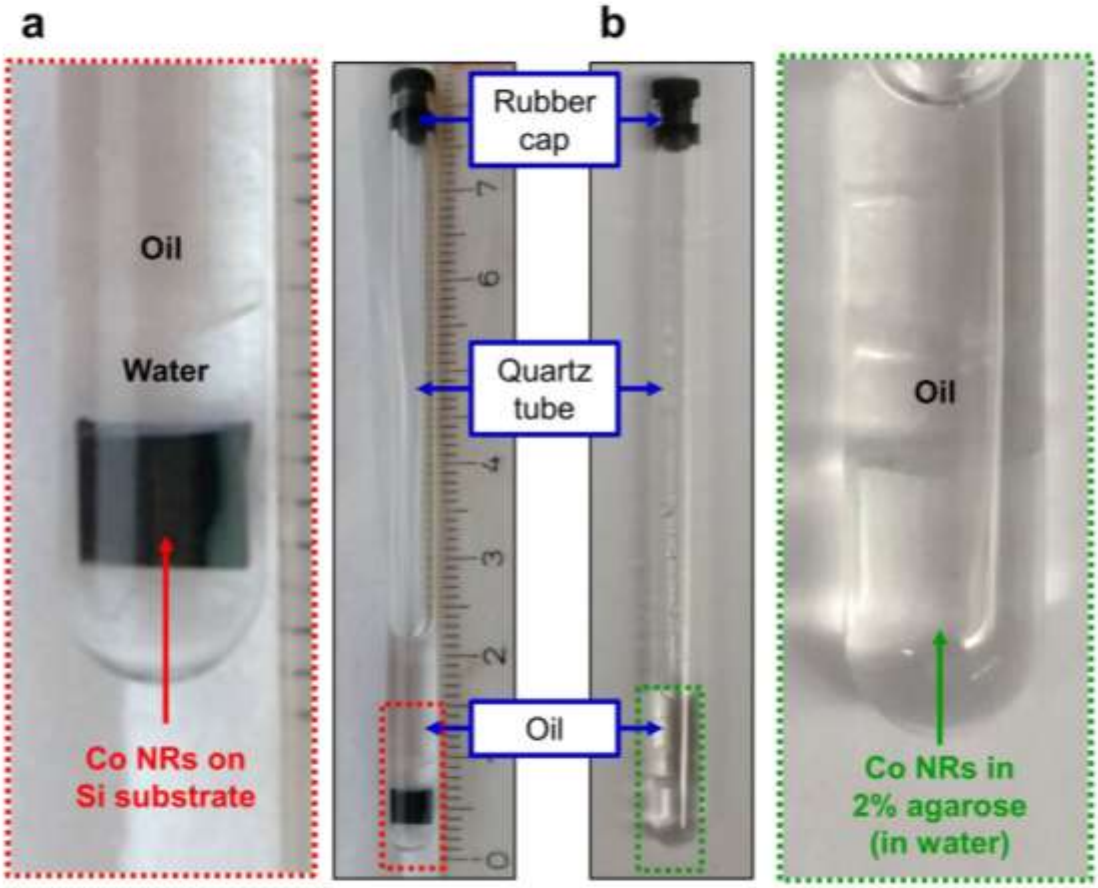

Figure S9. Sample preparation for *in situ* observations of ferromagnetic properties of Co NRs in water. a) A piece of Si wafer with the array of Co NRs was immersed in water in a quartz tube. To prevent water evaporation into the SQUID chamber, it was blocked by oil (left panel) and a 3D-printed rubber cap (right panel). b) At high temp., the colloidal solution of nanoparticles was mixed with 4% agarose and it was quenched in a fridge. Then, it was blocked by oil (right panel) and a 3D-printed rubber cap (left panel).

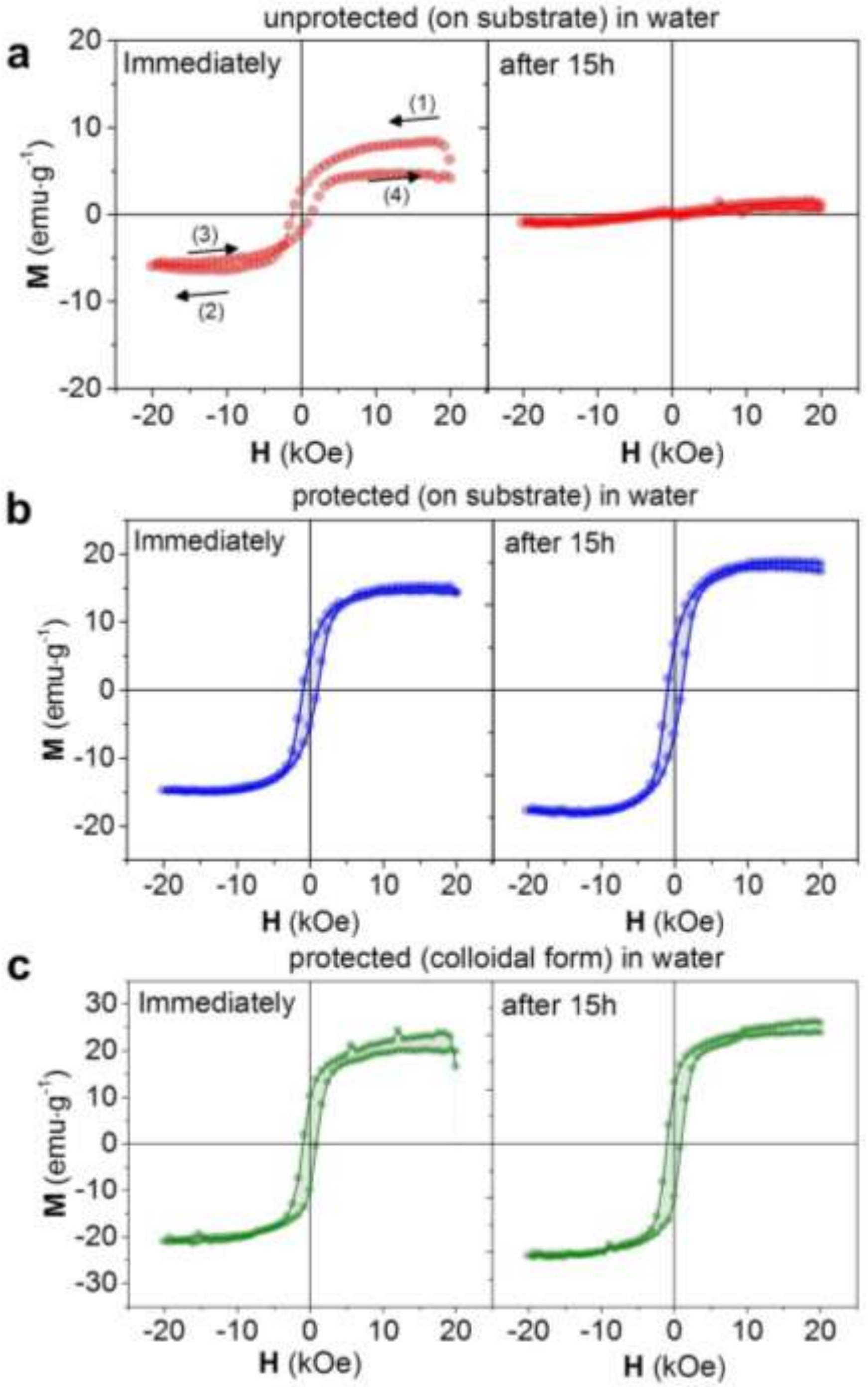

Figure S10. *In situ* observation of the ferromagnetic properties of the Co nanorods in water: In-plane magnetic properties of a) the unprotected Co nanorods and b) protected Co nanorods with 4 nm HfO_2 layer on the Si substrate in water (the numbers indicate the order of the measurements with the arrows showing the magnetic field sweep direction). c) Magnetic property of the protected Co nanorods with 4 nm HfO_2 layer suspended with the isotropic orientation in 2% agarose gel. Each panel on the left shows the measurement immediately after sample preparation and the right panels show measurements after the sample has been exposed to water for 15h.

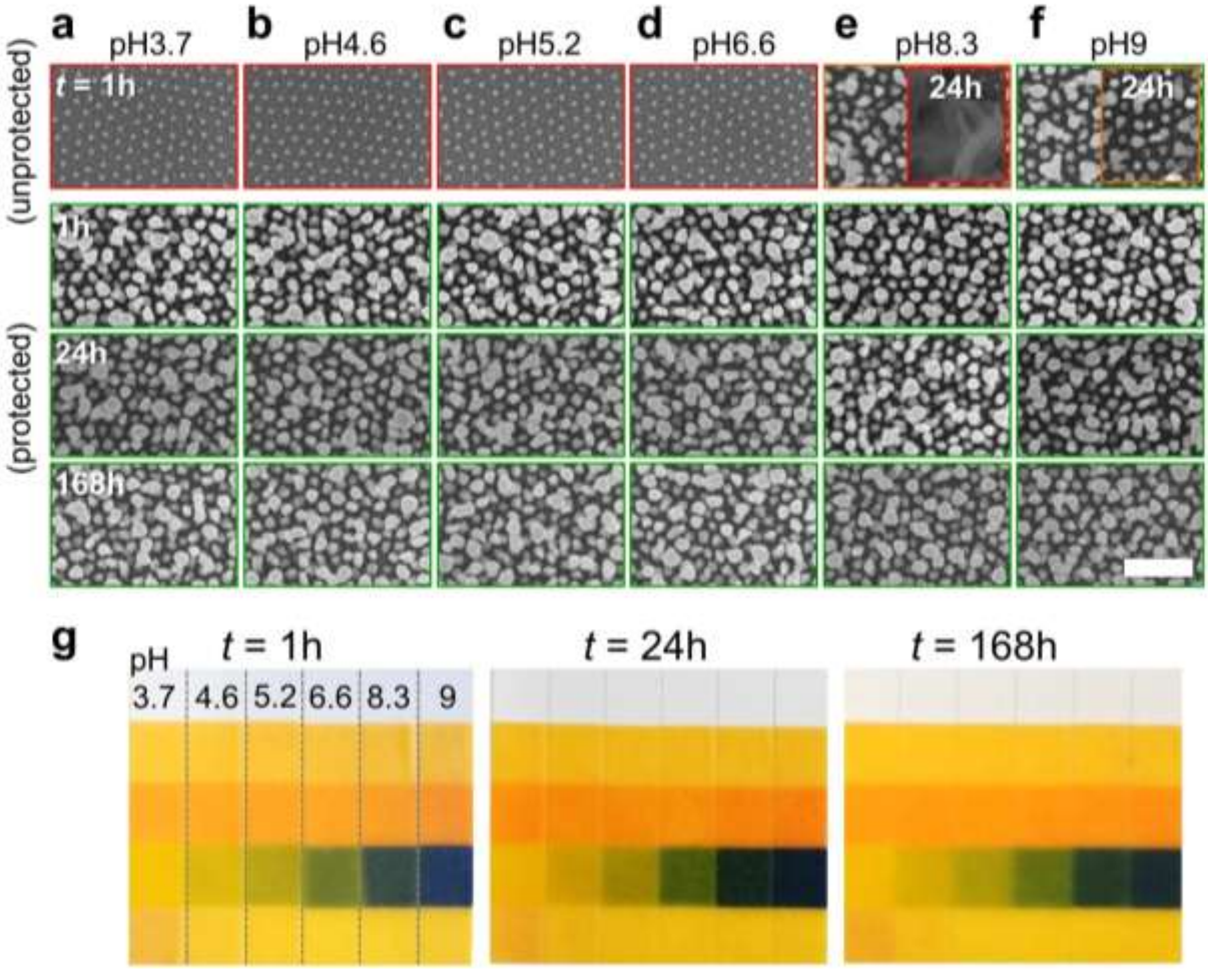

Figure S11. Stability of the protected Co nanorods in 0.1 M phosphate buffer solutions at a) pH 3.7, b) pH 4.6, c) pH 5.2, d) pH 6.6, e) pH 8.3, and f) pH 9. The first row shows the SEM images of the unprotected Co nanorods after 1h in the buffer solutions at different pH. The second to forth rows indicate the SEM images of the protected (encapsulated) Co nanorods with 4 nm HfO_2 layer after 1h, 24h, and 168h respectively in the buffer solutions at different pH (scale bar: 400 nm). g) The pH measurements of each buffer solution after 1h, 24h, and 168h (left to right: pH 3.7, 4.6, 5.2, 6.6, 8.3, and pH 9). The different boundary colors of each image indicate the status of the particles stability (red: complete corrosion, orange: partial corrosion, green: stable).

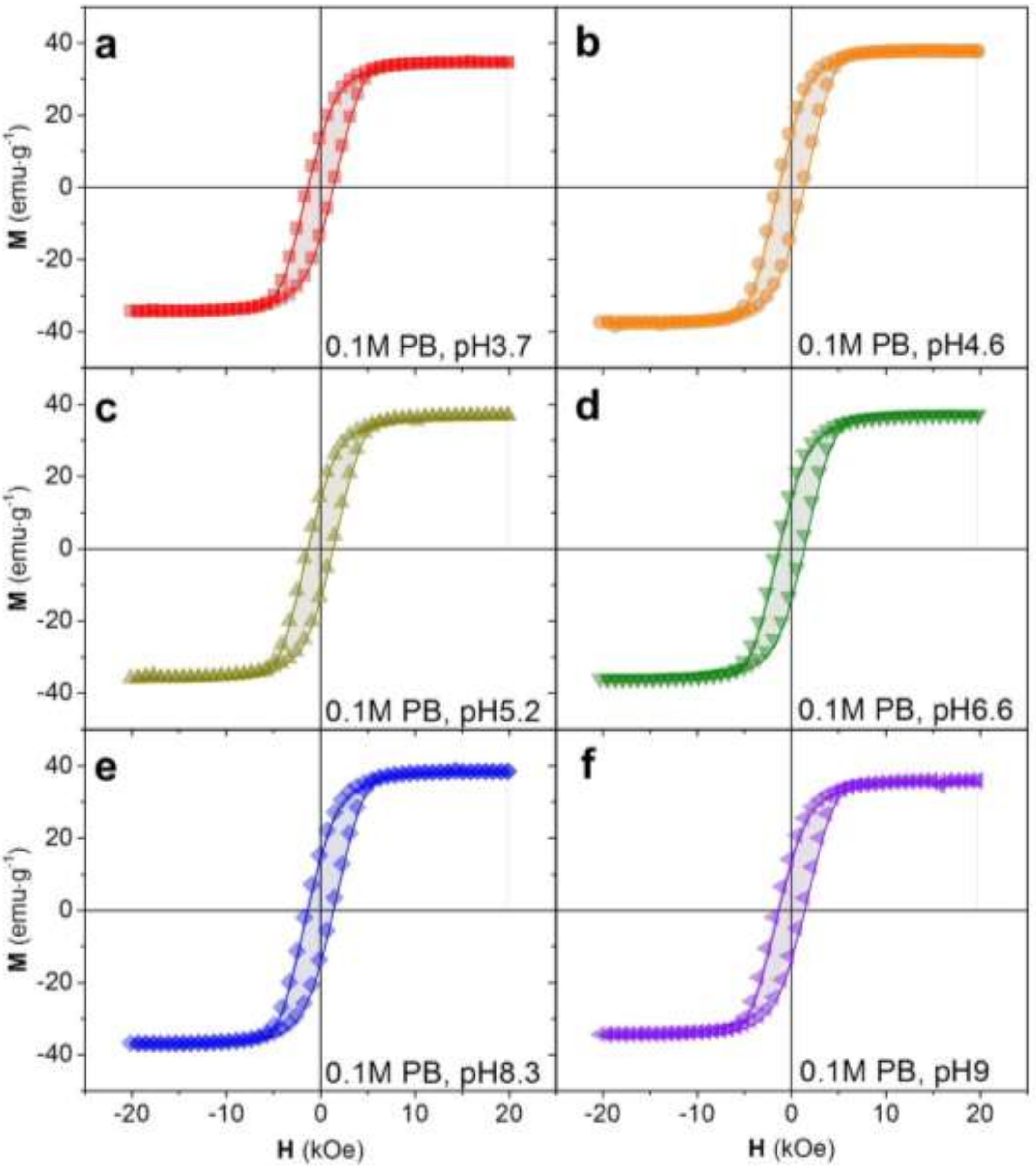

Figure S12. Out-of-plane ferromagnetic properties of the protected Co nanorods on the Si wafer after 7 days in 0.1 M phosphate buffer solutions at a) pH 3.7, b) pH 4.6, c) pH 5.2, d) pH 6.6, e) pH 8.3, and f) pH 9.

3. Cu nanorods protected with HfO_2

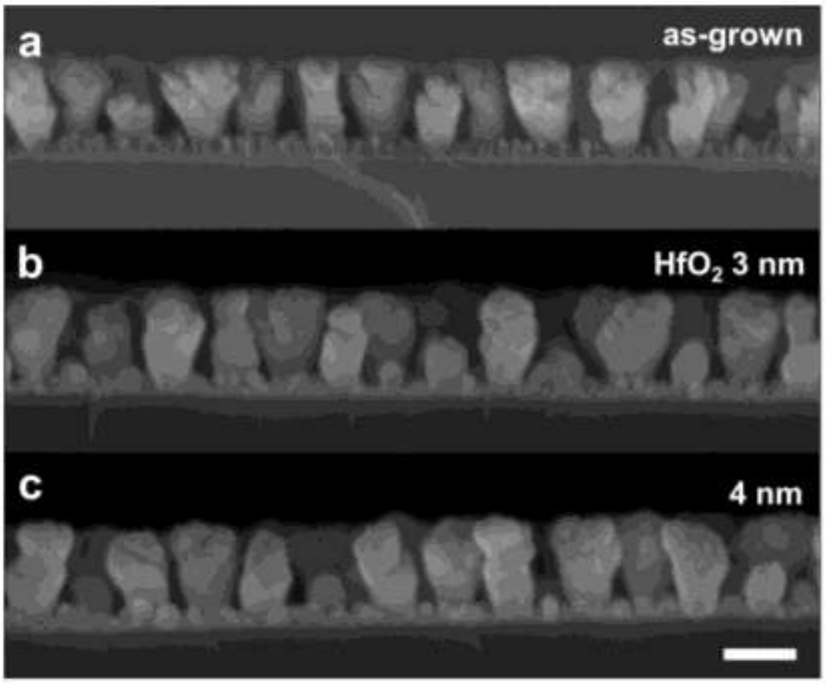

Figure S13. SEM images (side view) of the Cu nanorods protected with different thicknesses of the HfO_2 shell layer: a) 0 nm, b) 3 nm, and c) 4 nm (scale bar: 100 nm).

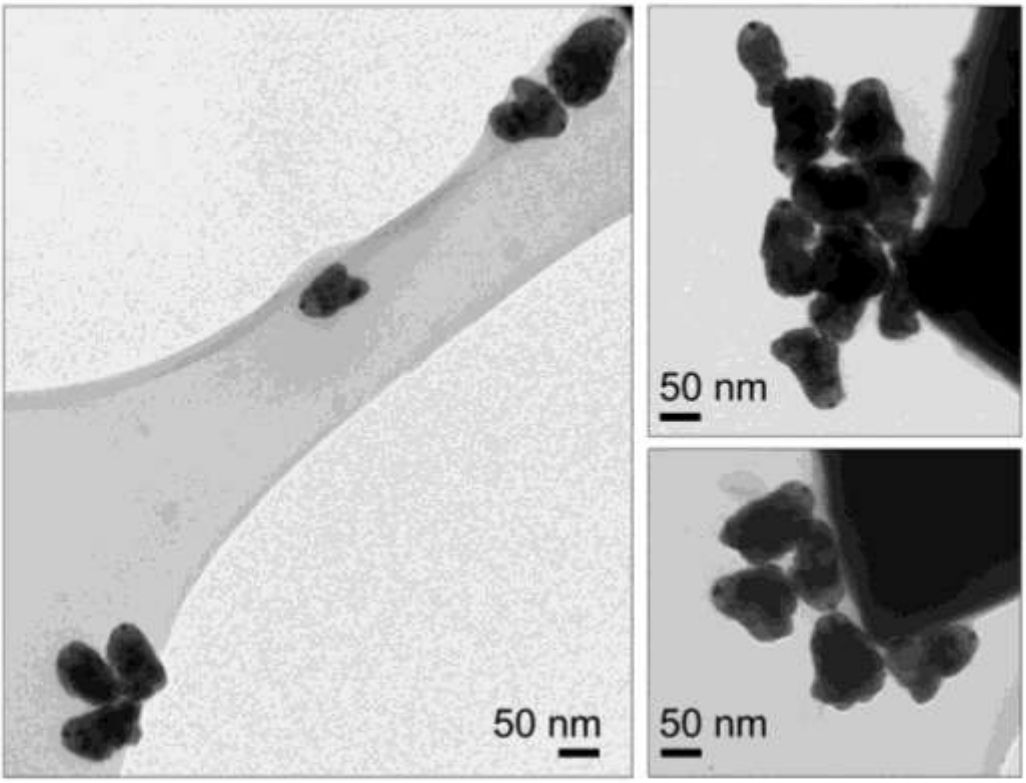

Figure S14. TEM images of the Co nanorods protected with 3 nm thick HfO_2 layer after 2 days in 0.1 M phosphate buffer solution at pH 3.7.

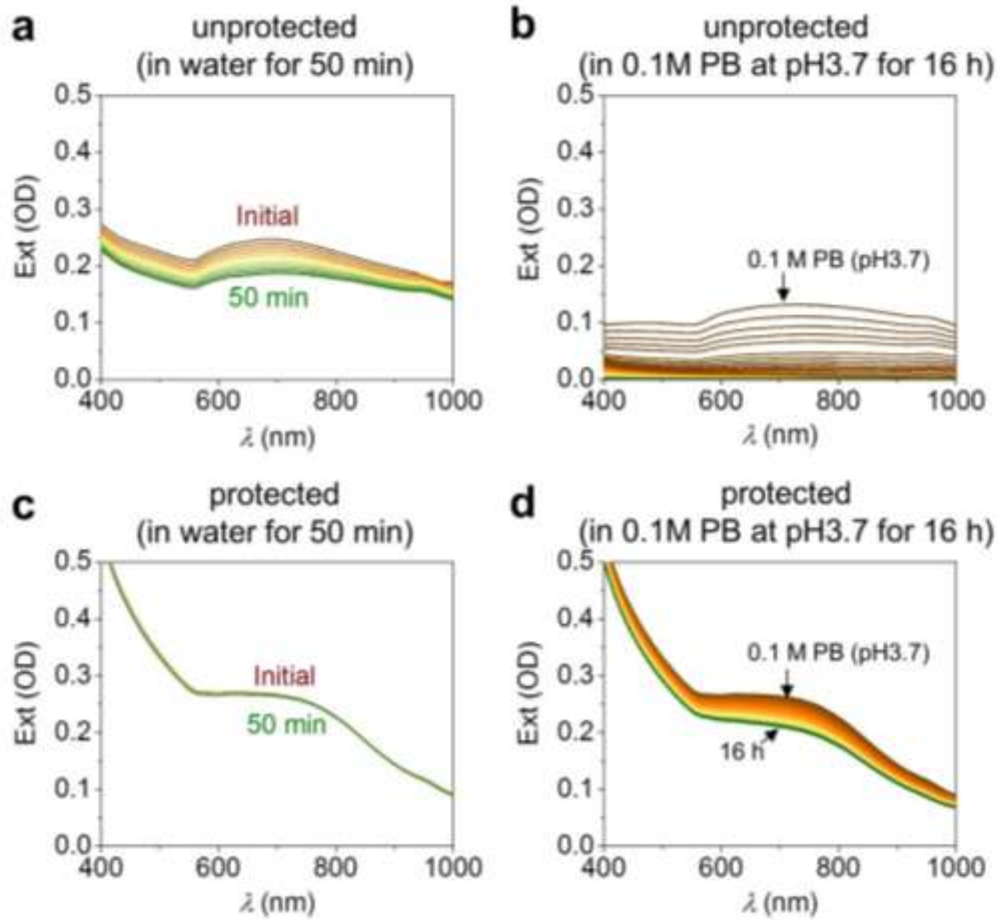

Figure S15. *In situ* observation of the stability of colloidal Cu nanorods in 0.1M phosphate buffer solution at pH 3.7. The extinction spectra of the unprotected Cu nanocolloids in a) 2% agarose gel for 50 min and b) after adding 0.1M phosphate buffer solution for 16 h with 5 min intervals. The extinction spectra of the protected Cu nanocolloids with a 4 nm HfO_2 layer in c) 2% agarose gel for 50 min and d) after adding 0.1M phosphate buffer solution for 16 h measured in 5 min intervals.

4. Cu nanohelices protected with Al_2O_3

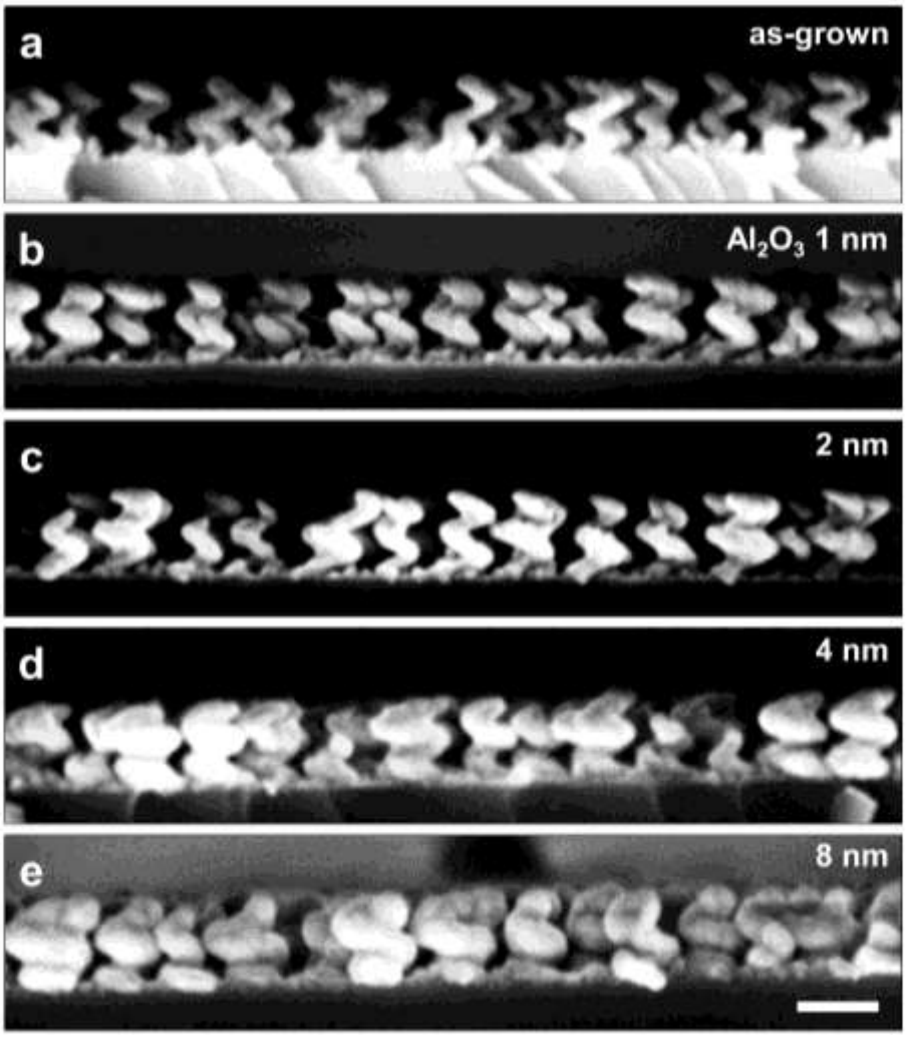

Figure S16. SEM images (side view) of Cu nanohelices with different thicknesses of the Al_2O_3 layer. a) 0 nm, b) 1 nm, c) 2 nm, d) 3 nm, and e) 4 nm (scale bar: 100 nm).

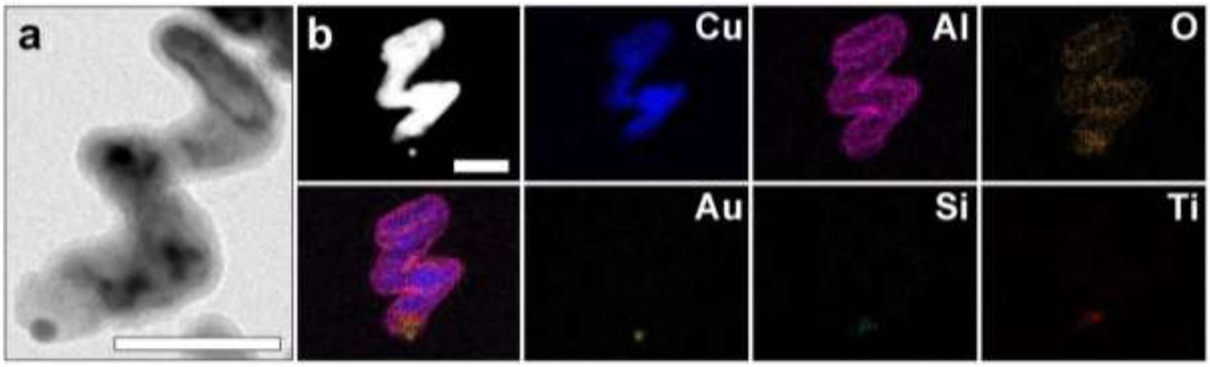

Figure S17. a) TEM image of single Cu nanohelix protected with 3 nm Al_2O_3 layer. b) Its corresponding STEM image and serial energy dispersive x-ray spectroscopy (EDX) false-color elemental maps.

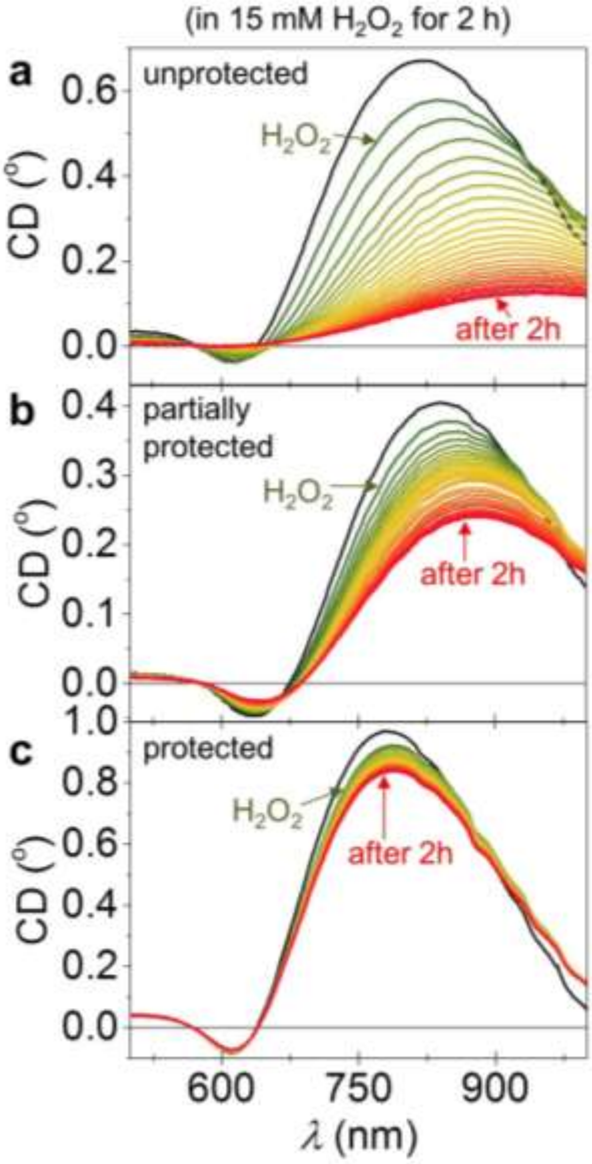

Figure S18. *In situ* observation of the chiroptical spectra of colloidal Cu nanohelices in 15 mM H_2O_2. a) unprotected (without plug and shell), b) partially protected (without plug), and c) completely protected (with plug and shell).

5. Cu nanohelices protected with HfO_2

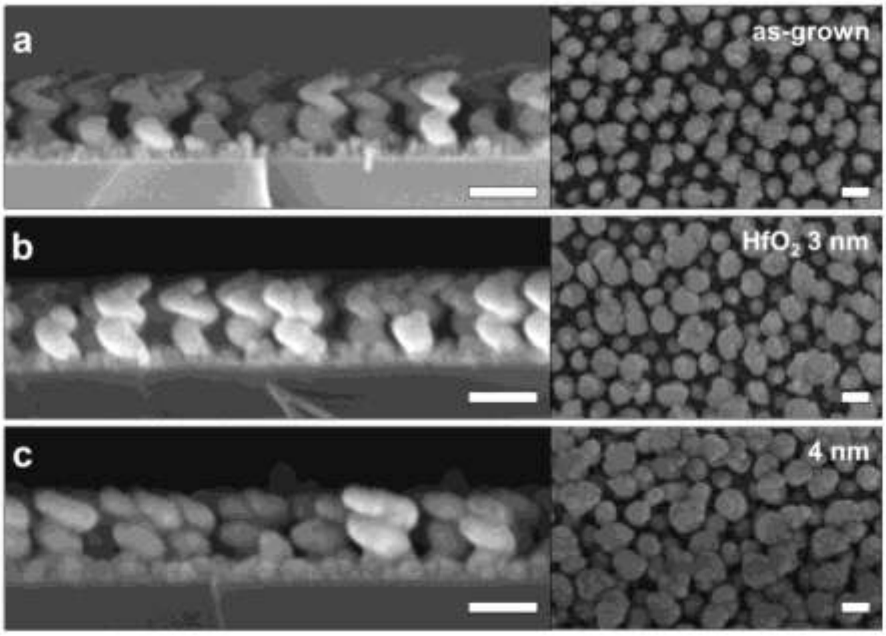

Figure S19. SEM images of the Cu nanohelices coated with different thicknesses of the HfO_2 layer. a) 0 nm, b) 3 nm, and c) 4 nm (Left panel: side view, right panel: top view, scale bar: 100 nm).

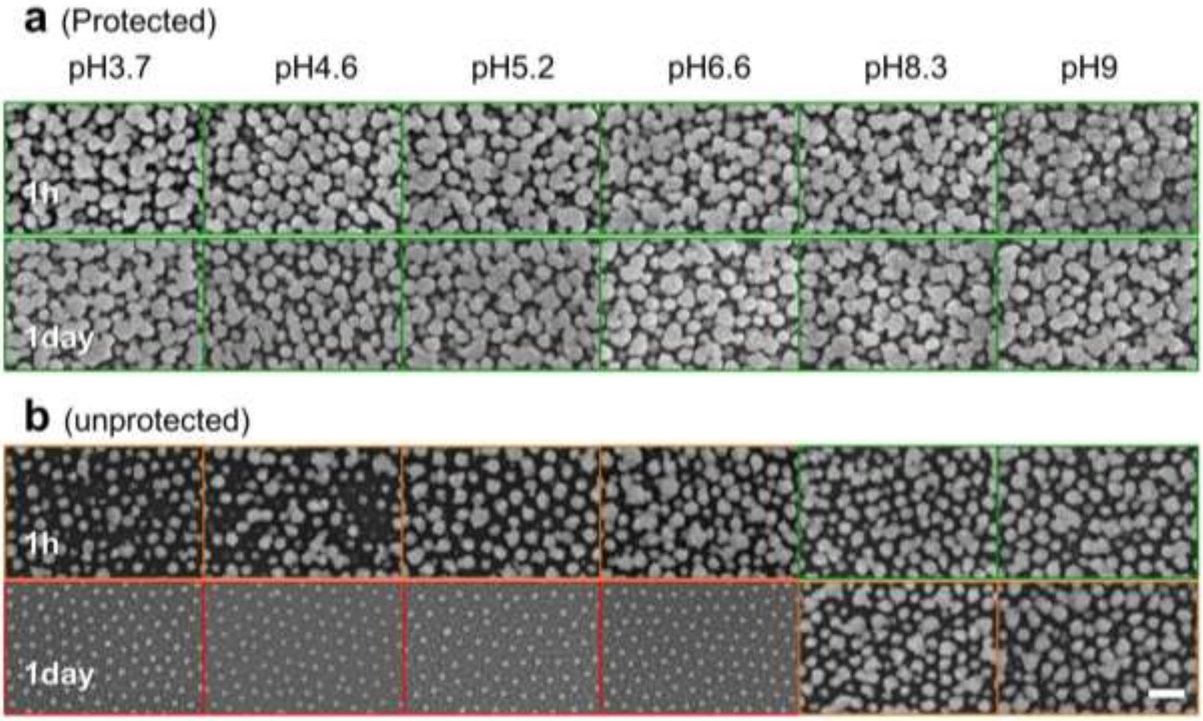

Figure S20. SEM images (top view) showing the stability of a) protected and b) unprotected Cu nanohelices in 0.1 M phosphate buffer solutions at pH 3.7, 4.6, 5.2, 6.6, 8.3, and 9 (scale bar: 200 nm). Each top panel is the resultant images after 1h in the buffer solution and each bottom panel is from the same sample after 1 day in solution. The different boundary colors of each image indicate the status of the particles stability (red: complete corrosion, orange: partial corrosion, green: stable).

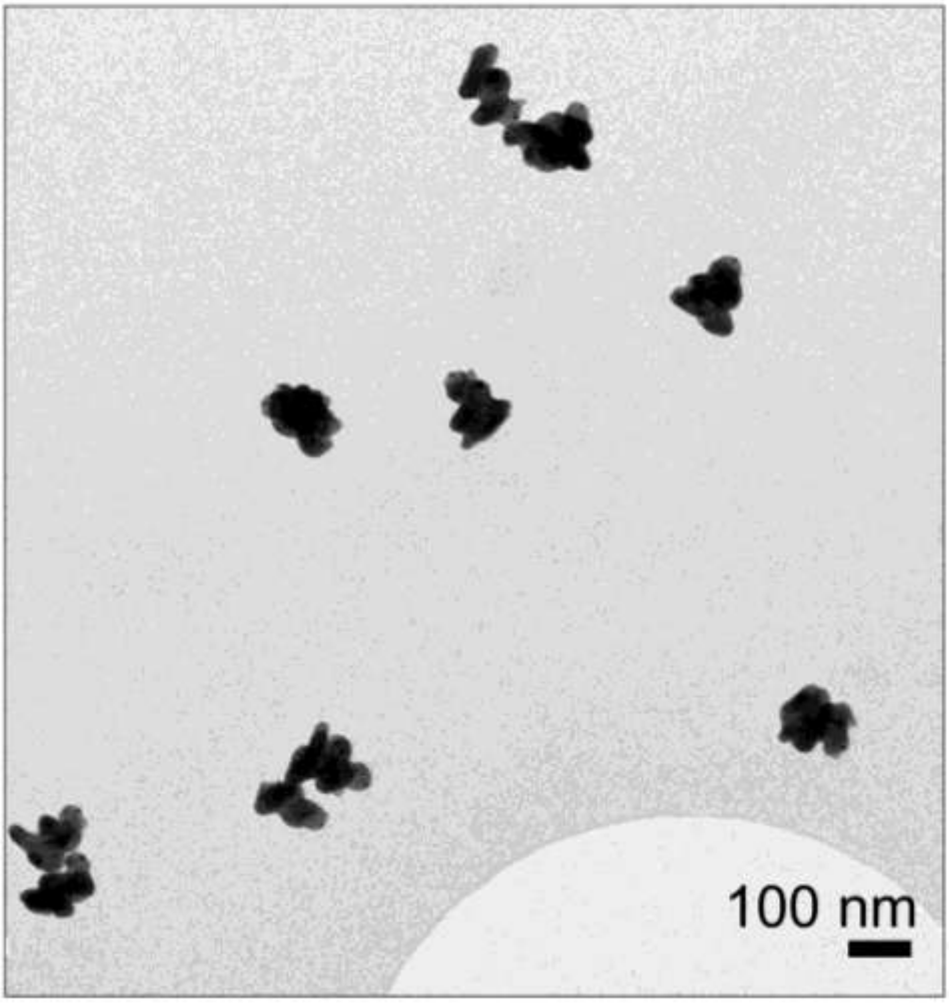

Figure S21. TEM image of the Cu nanohelices protected with 3 nm thick HfO_2 layer after 2 days in 0.1 M phosphate buffer solution at pH 3.7.

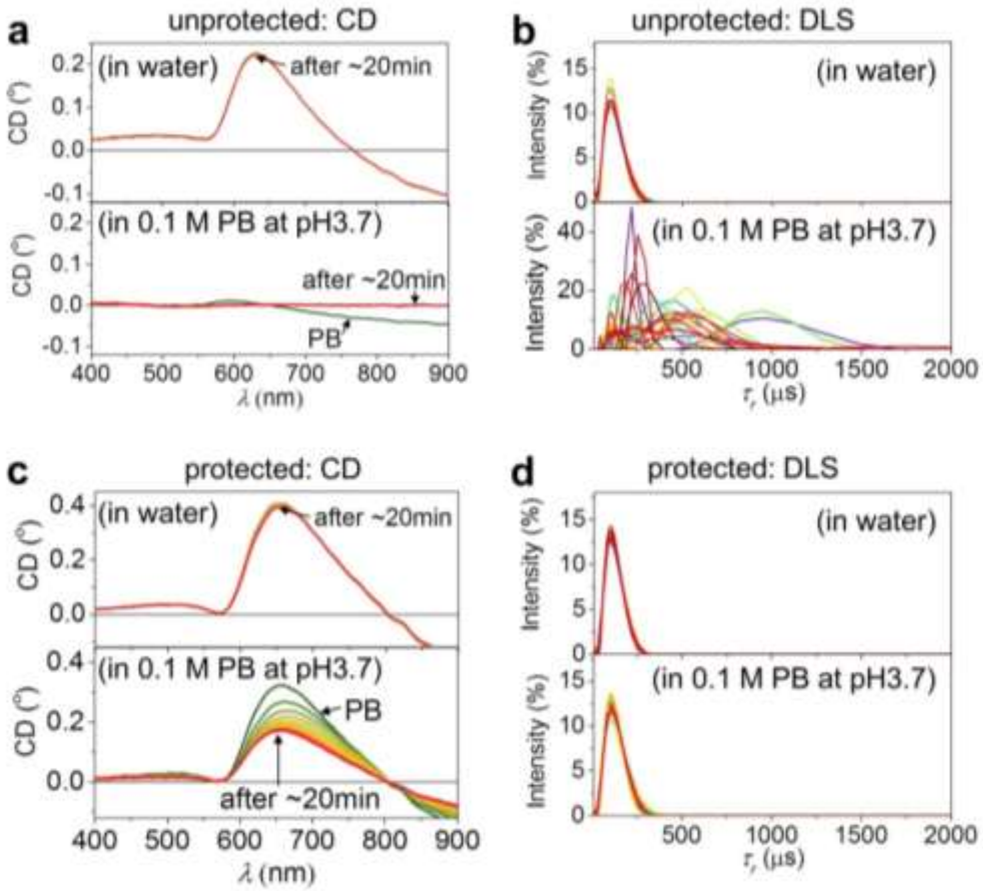

Figure S22. Stabilities of the unprotected and protected Cu nanohelices in 0.1 M phosphate buffer solutions at pH 3.7. a) The chiroptical spectra and b) DLS spectra of the colloidal unprotected Cu nanohelices in water (top panel) and buffer (bottom panel) for 20 min with 2 min intervals. c) The chiroptical spectra and d) DLS spectra of the colloidal protected Cu nanohelices in water (top panel) and buffer (bottom panel) for 20 min with 2 min intervals.

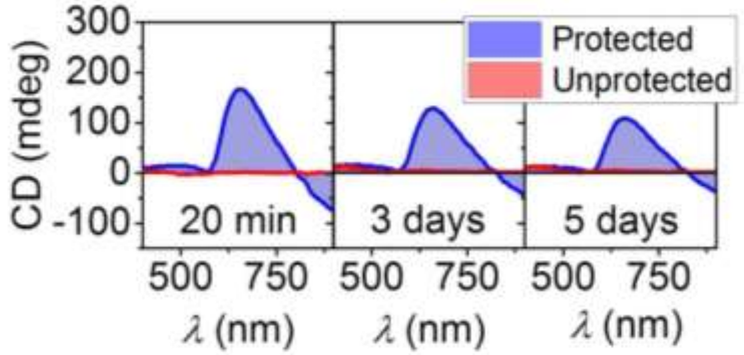

Figure S23. The chiroptical spectra of the colloidal unprotected Cu nanohelices in 0.1 M phosphate buffer at pH 3.7 after 20 min (left panel), 3 days (middle panel), and 5 days (right panel).

6. Corrosion kinetics

Corrosion reaction of metal nanoparticles can be simplified as, (ref. 7)

$$M(s) \rightarrow M^{2+} + 2e^{-}. \tag{1}$$

This reaction can be approximated as a first order reaction and the reaction decay in our measurements can be fitted by the exponential function

$$A = A_o \cdot \mathrm{e}^{-kt}, \tag{2}$$

where A is functional property of nanoparticles (i.e. magnetization, optical extinction, and circular dichroism), k is first order reaction rate, and t is reaction time. As copper ions do not give rise to an optical (plasmonic) signal, the decay rate of Cu nanoparticles (both rod and helix) can be readily estimated by Eqn. (2). However, Co ions possess a weak magnetic property (~2 emu/g, ref. 26), so that the Eqn. (2) should be extended as

$$A = A_o \cdot \mathrm{e}^{-kt} + A_o'(1 - \mathrm{e}^{-kt}), \tag{3}$$

which reflects the evolution of magnetization of Co ions in the second term. So, according to Eqns. (2) and (3), we calculated the decay rates of all the particles that are summarized in Table S1.

Table S1. Decay rates of unprotected Co nanorods, Cu nanorods, and Cu nanohelices in various different environments.

Nanoparticle	Protection	Solution	k [s^{-1}]	Reference
Co nanorod	unprotected	water (with 2% agarose)	142×10^{-6}	Figure 2c
Cu nanorod	unprotected	water (with 2% agarose)	92×10^{-6}	Figure 3c
Cu nanorod	unprotected	0.1M PB at pH3.7 (with 2% agarose)	563×10^{-6}	Figure 3c
Cu nanohelix	unprotected	10 mM H_2O_2	250×10^{-6}	Figure 4d
Cu nanohelix	partially protected	10 mM H_2O_2	63×10^{-6}	Figure 4d
